# Identification of NAA40 as a Potential Prognostic Marker for Aggressive Liver Cancer Subtypes

**DOI:** 10.3389/fonc.2021.691950

**Published:** 2021-06-02

**Authors:** Costas Koufaris, Antonis Kirmizis

**Affiliations:** Department of Biological Sciences, University of Cyprus, Nicosia, Cyprus

**Keywords:** protein N-terminal acetylation, hepatocellular carcinoma, the Cancer Genome Atlas (TCGA), histone modification, P53 signature, NAA40

## Abstract

Liver hepatocellular carcinoma (LIHC) is a leading cause of cancer-related mortality. In this study we initially interrogated the Cancer Genome Atlas (TCGA) dataset to determine the implication of N-terminal acetyltransferases (NATs), a family of enzymes that modify the N-terminus of the majority of eukaryotic proteins, in LIHC. This examination unveiled NAA40 as the NAT family member with the most prominent upregulation and significant disease prognosis for this cancer. Focusing on this enzyme, which selectively targets histone proteins, we show that its upregulation occurs from early stages of LIHC and is not specifically correlated with any established risk factors such as viral infection, obesity or alcoholic disease. Notably, in silico analysis of TCGA and other LIHC datasets found that expression of this epigenetic enzyme is associated with high proliferating, poorly differentiating and more aggressive LIHC subtypes. In particular, NAA40 upregulation was preferentially linked to mutational or non-mutational P53 functional inactivation. Accordingly, we observed that high NAA40 expression was associated with worse survival specifically in liver cancer patients with inactivated P53. These findings define NAA40 as a NAT with potentially oncogenic functions in LIHC and uncover its prognostic value for aggressive LIHC subtypes.

## Introduction

Liver cancer is one of the leading causes of cancer mortality worldwide, with more than 700,000 causing mortalities per year (1). This high mortality associated to liver cancer is due to late detection and its refractory nature to chemotherapy and/or surgical treatments. The most frequent type of primary cancers of this organ are liver hepatocellular carcinomas (LIHC), arising from malignant hepatocytes (1, 2). There are several known risk factors for LIHC, with the most prominent being hepatitis B/C infection, alcohol abuse, environmental chemicals such as aflatoxin, obesity, diabetes, and other metabolic diseases. The vast majority of liver cancers cases emerge within the context of chronic liver injury and inflammation (2). Importantly, epigenetic changes, most often elicited by environmental agents, are implicated in the initiation and progression of liver cancers (3, 4). In fact, a number of epigenetic drugs against liver cancer are in pre-clinical or clinical trials (5, 6). Based on these observations the investigation of the epigenetic mechanisms driving LIHC is considered a promising and important field of research for this tumour type.

Through substantial efforts of a number of research groups over the past two decades, it has also become possible to classify LIHC into molecularly and histologically distinct subtypes (7–11). One first major classification of liver cancers is their division into two approximately equal sized classes refer to as ‘proliferative’ and ‘non-proliferative’ (11). These two subclasses can be further subdivided into more refined molecular subclasses such as S1-S2 or G1-G3 corresponding to the proliferation class and S3 or G4-G6 for the non-proliferation class. LIHC subtypes differ in various aspects such as their underlying mutations, degrees of loss of hepatic differentiation, and disease prognosis (7–9, 11). It is important, therefore, to determine whether genetic mutations or epigenetic deregulations observed in LIHC are associated with specific subtypes of this cancer.

Acetyltransferases are enzymes that transfer acetyl groups to proteins, with this post-translational modification (PTM) potentially affecting the target proteins in multiple ways. The involvement of lysine acetyltransferases, which acetylate the lysine residue of histone and non-histone proteins, in LIHC has been extensively studied and is well established (12–14). Another category of protein acetyltransferases are the N-terminal acetyltransferases (NATs), that specifically modify the N-terminal α-amino group of proteins or polypeptides (15). Seven NATs and their catalytic subunits (NAA10-NAA80) have been identified and characterised in eukaryotes so far that differ in their evolutionary conservation, localisation, and target repertoire (16). N-terminal acetylation (Nt-Ac) of proteins affects their stability, localization and activity, ultimately impacting cell and organism function (16, 17). In fact, the majority of soluble eukaryotic proteins are Nt-Ac, although the biological significance of this modification has only started to be discovered relatively recently e.g. NatA was reported to modulate resistance to stress in plants (18), NatH/NAA80 is an actin-specific NAT that can affect cell motility and Golgi organisation (19, 20) and NatD/NAA40 controls gene expression and cellular lifespan in yeast (21, 22). Beyond the physiological functions of NATs, emerging studies have also reported their involvement in human diseases, especially cancers (23–28).

Over the past decade, researchers have gained access to multi-omic data coupled to patient clinical features for various cancer types as part of The Cancer Genome Atlas (TCGA) cohort. In a previous publication examining the NAT family across the TCGA cohort we had reported that the NAA40 mRNA is increased in multiple tumour types, with the most prominent upregulation and association with survival occurring for LIHC patients (27). Although we had noted this as a finding of potential significance, we had not examined in depth the association of NAA40 with LIHC previously e.g. if this change in NAA40 is associated with specific risk factors, genetic context or molecular subtypes. By performing a more thorough investigation of the TCGA and other independent LIHC cohorts in this study, we clarify the context of the deregulation of NAA40 in LIHC and its prognostic role in aggressive tumours with P53 mutations.

## Materials and Methods

Clinical, transcriptomic, and genomic data for liver samples in TCGA cohort were obtained through the Xena platform (29). For the TCGA LIHC cohort the transcriptomic data used were log_2_ (RSEM (RNA-Seq by Expectation-Maximization) +1). For each NAT gene the relative expression was calculated between the tumour and the normal groups and significance was tested by Student’s t-test. Patient clinical information were obtained when available from Xena platform or from **Supplementary Material** of original publication of TCGA LIHC cohort (30). For certain clinical characteristics information was not available for all samples within the TCGA LIHC cohort. NAA40 expression data (log_2_ (transcript per million (TPM)+1) for cancer cell lines were obtained from the Depmap project portal (https://depmap.org/portal/achilles). Cell lines originating from ten different tissues of origin were examined, ranging from 22-107 cell lines per tissue. For all other LIHC cohorts, cell or animal data examined in this study the transcriptomic data were obtained from GEO omnibus (31). Where multiple NAA40 probes were represented in arrays the mean value was subsequently used without any further normalization. Information on the studies examined and cohort sizes are tabulated in **Table 1**.

**Table 1:** Information on studies examined as part of this study.

Analysis	Cohort(s)	Cohort size
Comparison of NAT expression in tumour vs adjacent normal liver tissue and association with survival	TCGA	371 tumour and 50 normal samples
GSEA comparison of top and bottom quartiles of NAA40 expression in LIHC tumours	TCGA; GSE112790.	TCGA 90 samples for top and bottom quartiles; GSE112790 46 samples for top and bottom quartiles
LIHC disease stage	TCGA	50 samples for the adjacent normal group, Stage I consisted of 150 samples, Stage II of 98 samples, and Stage III of 97 samples
Obese and non-obese LIHC patients	TCGA	127 obese patients and 37 non-obese
LIHC patients with cirrhosis vs no cirrhosis	TCGA	47 cirrhotic patients and 140 non-cirrhotic patients
LIHC patients with stages of liver inflammation	TCGA	117 were defined as non-inflamed, 99 as mild inflammation, and 18 as severely inflamed
LIHC patients at different Fibrosis stages	TCGA	97 were classified as stage 0, for stages 1&2 35 samples, stages 3&4 31, stage 5 10 samples, and for stage 6 76 samples
LIHC patients with serological data for Hepatitis B or C infection	TCGA	207 serologically negative for both hepatitis B and C; 84 serologically positive for both hepatitis B and C; 60 positive only for hepatitis B and 19 only for hepatitis C
iCLuster classification	TCGA	65 iCluster 1, 55 iCluster 2, and 63 for iCluster 3
*P53* and *CTNNB1* mutation cohorts	TCGA	*P53* but no *CTNNB1* mutations (N=88); *CTNNB1* but no *P53* mutations (N=71); no identified mutations in either of the two genes (N=171)
Association of NAA40 with survival and P53 activity	GSE14520; GSE54236	GSE14520 221 samples with survival and transcriptomic data; GSE54236 78 samples with survival and transcriptomic data
Hepatic differentiation	GSE23034; GSE61287; GSE19044; GSE73617	GSE23034 compared hepatocytes (N=3) and hepatocyte derived induced pluripotent stem cells (iPSC) (N=3); GSE61287 comparison of hepatocytes, iPSC derived from hepatocytes, and hepatocyte-like cells (HLC) differentiated from the iPSC (N=1 for all groups); GSE19044 Germline cell-derived pluripotent stem cells (GPSCs) and Embryonic stem cells (ES) in naïve state or differentiated into hepatocytes (N=2 all groups); GSE73617 Mesenchymal stromal cells (MSCs), HLC derived from MSC, and HLC induced to dedifferentiate (N=3 each group
P53 manipulation	GSE34760; GSE30137; GSE30137	GSE34760 compared liver tumours derived from p53 KO mice and from p53 wildtype liver tumours induced by N-nitrosodiethylamine (DEN) treatment (N=4-6 per group); GSE30137 HepG2 cells treated with DMSO, shP53, Nutlin, Nutlin+shP53 (N=2 per group); GSE30137 acute knockout of P53 using adenoviral construct (N=3 for GFP control and CRE treatment respectively)
Cancer cell lines	DepMap	Cell lines originating from ten different tissues of origin were examined, ranging from 22-107 cell lines per tissue.

GSEA (32) was performed on two LIHC cohorts (TCGA 371 LIHC samples and GSE112790 183 samples) that were divided into top and bottom quartiles according to their NAA40 expression. 157 Liver related datasets were downloaded from MSigDb (29), of which 131 genesets were retained for further analysis because they contained between 15-500 genes. After completion of 1000 permutations significant genesets were considered those with nominal p.val <0.05 and FDR q<0.25, as recommended in the GSEA user guide (https://www.gsea-msigdb.org/gsea/doc/GSEAUserGuideFrame.html).

To investigate the association between NAA40 expression and LIHC patients overall survival (OS), gene expression, and exon mutation data for TCGA LIHC cohort were obtained through Xena platform. For the first analysis patients were grouped according to mutations into those with *P53* but no *CTNNB1* mutations (N=88), *CTNNB1* but no *P53* mutations (N=71), and those with no identified mutations in either of the two genes (N=171). A second survival analysis was performed on TCGA LIHC samples with the 171 samples with no *P53/CTNNB1* mutations. For these patients, their p53 functionality was ranked using the p53 signature as described previously and then sub-divided into the high P53 functionality and the low P53 functionality groups (N=85 each group). For each group patients were divided according to median NAA40 expression and Kaplan-Meier survival curves were plotted. Hazard Ratios (HR) and Statistical significance were calculated using the log-rank method with significance set at p<0.05. For the analysis of the association of NAT family in TCGA LIHC the oncolnc tool was used (33). Heatmaps were generated using the Morpheus software tool (https://software.broadinstitute.org/morpheus) with genes after Robust Z-transformation and clustering by Euclidian metric and average linkage.

The P53 activity predicting signature of 10 repressed genes was defined previously (*CCNB1*, *PLK1*, *EED*, *CDK1*, *EZH2*, *CCNB2*, *E2F3*, *MYBL2*, *FOXM1*, *E2F2*) (30). To define LIHC samples as low or high P53 activity the levels of each of these 10 genes was ranked in relevant samples, a composite score was then calculated by adding the rank values, and finally the samples were ranked as higher or lower p53 functionality based on this score.

## Results

### NAA40 Is the Most Upregulated and Only NAT Correlating With Survival Outcome in LIHC

In order to gain further insight on the potential association of NATs with LIHC we performed a multi-omic investigation of NATs in the TCGA pan-cancer study as we had done previously (27), but focusing specifically on this tumour type. Specifically, we examined in the TCGA LIHC cohort (1) their mutational frequencies; (2) transcript levels in adjacent normal and primary tumour tissue; and (3) association of their expression with disease prognosis. Regarding genomic alterations (copy number alterations and exon mutations) these were generally rare events for NAT genes in LIHC (0-3% range, **Figure S1**). Comparison of transcript levels found that in this patient cohort NAA40 was the NAT enzyme with the greatest differential expression between non-tumour and tumour samples (2.1 Fold change, p<0.001 **Table 2**), with NAA10 and NAA20 also upregulated more than 1.5-fold in tumour samples. For survival analysis LIHC patients were split into high or low groups for each NAT based on corresponding median gene expression. Kaplan-Meier plots were derived and groups were tested for significant differences using the logrank test. Notably, based on this analysis only NAA40 was significantly associated with patient survival (Logrank p-value=0.003; all Kaplan-Meier plots can be seen **Figure S2**). Together, the analyses on survival and transcript levels highlight NAA40 as the NAT with the most significant association in LIHC.

**Table 2:** Comparison of NAT expression levels between adjacent normal and primary tumour tissue in TCGA LIHC dataset.

Gene	Adjacent Normal	Primary Tumour	Fold Change	p. value
NAA40	6.9 ± 0.4	8.0 ± 0.8	2.1	p<0.01
NAA10	9.6 ± 0.3	10.4 ± 0.7	1.7	p<0.01
NAA20	10.2 ± 0.2	10.8 ± 0.6	1.5	p<0.01
NAA80	7.3 ± 0.6	7.8 ± 0.8	1.4	p<0.01
NAA25	8.0 ± 0.4	8.4 ± 0.6	1.3	p<0.01
NAA60	10.9 ± 0.2	11.2 ± 0.4	1.2	p<0.01
HYPK	10.6 ± 0.4	10.7 ± 0.7	1.1	N.S.
NAA15	9.3 ± 0.5	9.3 ± 0.5	1.0	N.S.
NAA35	7.9 ± 0.6	7.9 ± 0.4	1.0	N.S.
NAA50	11.1 ± 0.2	11.1 ± 0.4	1.0	N.S.
NAA30	9.4 ± 0.3	9.0 ± 0.5	0.7	p<0.01

Genes are arranged in descending order according to Fold-change between adjacent normal and tumour tissue. Values shown are Pan-cancer normalised values+1 and standard deviation. Statistical significance was tested by Student’s t-test with Bonferroni multiple testing correction.

Considering the above connection, it was important to examine whether the upregulation in NAA40 transcript levels is reflective of a corresponding increase in protein levels. Due to the fact that proteomic data for NAA40 were not available from the TCGA cohort, we examined the DepMap project (https://depmap.org/portal/), for which transcriptomic and quantitative proteomic data were available. Across a panel of 14 liver cell lines there was a strong correlation between transcript and protein measurements for NAA40 (Pearson’s correlation r=0.79), suggesting that the observed increase in mRNA levels of NAA40 in LIHC is coupled to an increase in its protein levels (**Figure S3**).

Thus, amongst all NATs, NAA40 displays the highest potential for playing a role in LIHC as indicated by its significant transcriptional upregulation and concomitant association with survival in the TCGA patient cohort.

### NAA40 Upregulation in LIHC Occurs From Early Malignant Stages Irrespective of the Underlying Etiological Factors

**Figure 1:**
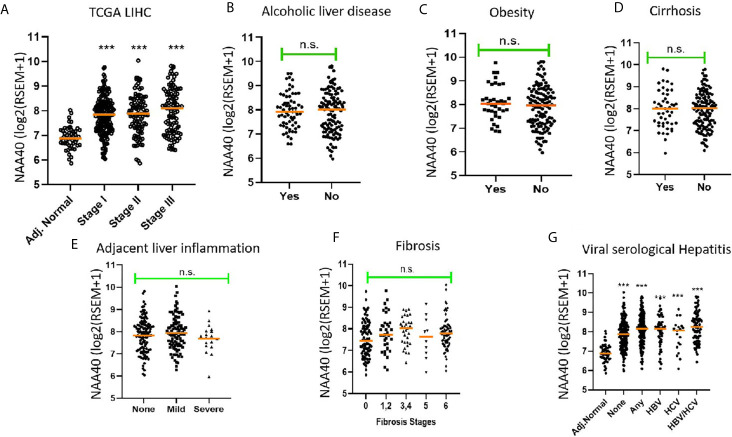
Association of NAA40 expression with LIHC tumour stages and exposure to specific risk factors **(A)** NAA40 transcript levels in adjacent normal and stages I-III of LIHC derived from the TCGA cohort. Stage IV is not shown due to small sample size; **(B–G)** Association between NAA40 transcript levels and various etiological factors of LIHC. In the scatterplots each circle represents an individual sample and orange line depicts the median value. NAA40 expression values shown are pan-cancer normalised log2(RSEM+1). For panel **(G)** Adjacent normal (Adj.norm) refers to non-cancer tissue sampled adjacent to the tumour tissue, none refers to LIHC tumour samples with no serological evidence of viral infection, any refers to LIHC samples with serological evidence with infection with either one or both hepatitis B and C Statistical significance was tested by Student’s t-test or one-way ANOVA ***p < 0.01. n.s., non significant.

Since NAA40 emerged as the most promising candidate NAT for a role in LIHC, we then decided to focus further on this enzyme and clarify the drivers of its deregulation. It has been previously reported that upregulation of NAA40 occurs from early stages of colorectal cancer (28), but not of lung cancer where it is mainly associated with more advanced tumour stages (34). To determine what the case is for LIHC, we screened the TCGA LIHC dataset which contains samples from early to advanced cancer stages. It was apparent that the increase in NAA40 transcript levels is an early and persistent event during liver cancer progression (**Figure 1A**). For the most advanced stage IV there were fewer LIHC samples in the TCGA dataset and, therefore, the observed lower levels might be unreliable due to sampling error. Given the increased expression of NAA40 from Stage I LIHC, we then considered the possibility that this molecular event could also be occurring in pre-neoplastic tissue, perhaps driven by conditions such as chronic inflammation. Based on this we examined publicly available data from non-cancer liver studies. However, we did not observe any prominent differences for NAA40 transcript levels in non-malignant fibrotic settings (GSE89632 - 9.9 ± 0.4 in healthy vs 9.8 ± 0.4 non-alcoholic steatohepatitis (NASH) biopsies; GSE49541 – 3.5 ± 0.2 and 3.6 ± 0.3 in mild and advanced non-alcoholic fatty liver disease (NAFLD) respectively; GSE84044 - HBV liver fibrosis patients with Scheuer Grade “g” of inflammation 0-1 3.0 ± 0.2, grades 2-4 3.0 ± 0.3).

Next, we examined the association of NAA40 expression in LIHC with various known liver cancer etiological factors. Notably, there was no significant association of NAA40 with cirrhosis, alcoholic liver disease, obesity, fibrosis, inflammation or hepatitis infection (**Figures 1B–F**). The only significant difference found was between tumours with serological viral infection versus those without (**Figure 1F**), although the difference was modest (1.2 fold; p<0.05). However, compared to adjacent normal liver, a significant upregulation of NAA40 transcript was observed in all tumour samples regardless of the infecting agent (**Figure 1F**), consistent with the general upregulation of NAA40 in LIHC.

Collectively, the examined data support that NAA40 upregulation does not occur in premalignant lesions but emerges early in hepatocarcinogenesis and is not correlated with specific etiological factors.

### NAA40 Expression Has an Inverse Correlation With Hepatocyte Differentiation

**Figure 2:**
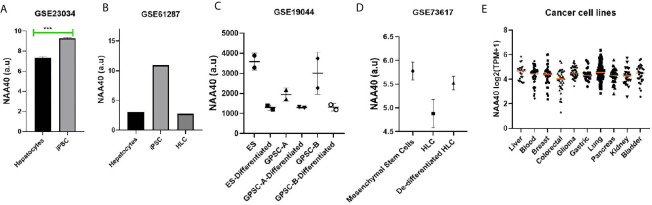
NAA40 transcript levels inversely correlated with hepatocyte differentiation **(A)** NAA40 levels in primary hepatocytes before and after de-defferentiation into iPSC; **(B)** NAA40 transcript levels in another study similar to **(A)** but including expression data from iPSCs that are differentiated again into hepatic-like cells (HLC); **(C)** Expression of NAA40 in Embryonic Stem Cells (ES) and Germ line cell-derived pluripotent stem cells (GPSC) in untreated state and after 28 day treatment to induce differentiation into hepatic-like cells; **(D)** NAA40 expression in resting mesenchymal stromal cells, mesenchymal stem cells induced to differentiate into HLC and after these are dedifferentiated again; **(E)** NAA40 expression across cancer cell lines originating from various human organs. Each circle represents a different cell line. Orange line indicated the median expression for cells of a given tissue of origin. Arbitrary Units (A.U) refers to normalised microarray values without any further processing. ***p < 0.001.

In a previous study, we observed that NAA40 transcript levels in the liver were among the lowest in the human body (27). Loss of hepatocyte differentiation is known to be a common event in LIHC and is associated with worse prognosis (35–37). Based on this we considered the possibility that the increased NAA40 levels could be related to the loss of hepatocyte differentiation. Accordingly, we found that NAA40 levels were increased by dedifferentiation of hepatocytes into induced pluripotent stem cells (iPSC), while conversely its levels decreased upon differentiation of iPSC into hepatic-like cells (HLC) (**Figures 2A, B**). A similar trend of reduced NAA40 levels was observed after differentiation of embryonic, germ line, and mesenchymal stem cells (**Figures 2C, D**). It is also interesting to note that NAA40 shows a negative correlation with hepatic differentiation not only in different cell types, but also across human (**Figures 2A, B**) and mouse (**Figures 2C, D**) cells, in agreement with the evolutionarily conserved function of NAA40 (38, 39). In line with this, monolayer-cultured liver cancer cells in which NAA40 is highly expressed are well-known to loose characteristic gene markers and metabolic properties of mature hepatocytes (11). We also noted that expression of NAA40 in liver cancer cell lines was comparable to cell lines derived from other tumour types (**Figure 2E**), unlike in tissues derived from normal organs where its expression differs significantly (27).

In conclusion, the analysis of available data is consistent with a negative correlation between NAA40 expression and hepatocyte differentiation.

### High NAA40 Expression Is Associated With More Aggressive Liver Cancer Subtypes

**Figure 3:**
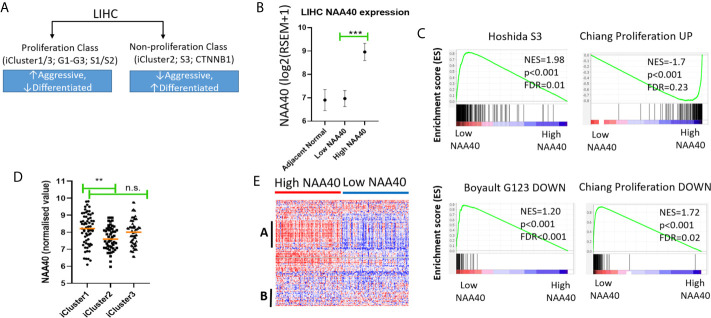
High NAA40 expression in LIHC is a characteristic of more proliferating and aggressive cancer subtypes. **(A)** Diagram depicting the major molecular subclasses of LIHC adapted from (11); **(B)** Comparison of NAA40 expression levels in adjacent normal tissue, bottom and top quartiles of TCGA LIHC dataset; **(C)** GSEA plots displaying enrichment of Hoshida_S3, Boyault_G123_DOWN and Chiang_proliferation_DOWN genesets in low NAA40 expressing samples and Chiang-proliferation_UP in the high expressing NAA40 samples; **(D)** Heatmap displaying expression of 125 cell cycle-associated genes in High and Low NAA40 samples, with red colour signifying high expression (>value) and blue colour low expression (<value)?. please check if this s/b captured as a panel and appeared as **(A, B)** indicate two predominant clusters with genes having a direct correlation **(A)** or inverse relationship **(B)** to NAA40 expression levels; **(E)** Expression of NAA40 in TCGA LIHC iClusters. In the scatterplots each circle represents an individual sample and orange line depicts the median value **p < 0.01, ***p < 0.001 Student’s t-test. n.s., non significant.

Our analysis so far indicated that NAA40 in LIHC is not associated with specific etiological factors, but could be related to loss of hepatic differentiation. A major established molecular classification of LIHC consists of two classes; one proliferating class characterised by more aggressive, less differentiating tumours and a non-proliferating class consisting of less aggressive, more differentiated tumours (11) (**Figure 3A**). Hence, we next examined whether NAA40 upregulation is correlated with the less well-differentiated, more aggressive subtype of LIHC. For this purpose we performed Gene Set Enrichment Analysis (GSEA) to gain insights into the molecular subtypes associated with NAA40 deregulation. First, TCGA liver cancer samples were divided into tumours with high-NAA40 expression (top quartile) and low-NAA40 expression (low quartile). It is noteworthy that there existed a significant difference in the expression of NAA40 between the High and Low groups (average 4-fold), with expression in the lower quartile being similar to that of adjacent normal tissue (**Figure 3B**). This indicates that there is a substantial variation in expression of this gene among liver tumours samples. Consistent with this notion, analysis of the high and low NAA40 expression groups by GSEA found that out of 131 examined liver genesets only 24 yielded significant results (nom. p.val<0.05; FDR q<0.25) (**Supplementary Tables 1, 2**), demonstrating that NAA40 upregulation is not homogeneous across all LIHC subtypes.

In regards to the major classification of LIHC into the proliferation and non-proliferation groups, GSEA indicated that NAA40 upregulation is primarily enriched in the former class (**Figure 3C**). Specifically, the gene signatures corresponding to high NAA40 expression in LIHC were enriched for proliferation-associated signatures (e.g. Chiang’s proliferation geneset), while low NAA40 was conversely significantly associated with non-proliferation signatures such as the Hoshida S3 geneset and Boyalt’s G1-G3 subclasses (selected genesets can be seen in **Figure 3C** and **Table 3**). Moreover, enriched gene signatures in samples showing high NAA40 expression were also ones indicative of worse prognosis (e.g. WOO_Liver_Cancer_Recurrence_DN; Hoshida_Liver_cancer_Survival_DN; LEE_Liver_Cancer_Survival_UP) consistent with previous observations (**Figure S2**). The TCGA LIHC study also reported the division of LIHC into three subtypes, iCluster 1-3 based on the integration of mRNA expression, DNA methylation, DNA mutation, miRNA, and RRPA platforms (30), which distinctly classify within the proliferation and non-proliferation classes (**Figure 3A**). Consistent with the observations above, the expression of NAA40 was significantly lower in samples classified in iCluster 2 (**Figure 3D**), which are less aggressive tumours and belong in the non-proliferation class. However, we also note that NAA40 levels were similar in iClusters 1 and 3, although iCluster 1 has overall a worse prognosis than the other two iClusters (30), a finding that is incongruent to a degree with the identified association of NAA40 with more aggressiveness and worse survival. Although it is not clear presently why NAA40 expression is high in both iCluster 1 and 3, it could be related to the highest frequency of P53 mutations in the later subgroup [see (30) and association with P53 in next section].

**Table 3:** Selected enriched genesets according to NAA40 levels relating to molecular classification of HCC.

Gene Set Name	NES	Nom. p.val	FDR q Val
HOSHIDA_LIVER_CANCER_SUBCLASS_S3	1.98	>0.0001	0.013
BOYAULT_LIVER_CANCER_SUBCLASS_G1_DN	1.79	0.008	0.017
ANDERSEN_LIVER_CANCER_KRT19_DN	1.83	>0.0001	0.019
BOYAULT_LIVER_CANCER_SUBCLASS_G123_DN	1.75	>0.0001	0.02
CHIANG_LIVER_CANCER_SUBCLASS_PROLIFERATION_DN	1.72	>0.0001	0.022
CHIANG_LIVER_CANCER_SUBCLASS_CTNNB1_UP	1.72	0.02	0.023
BOYAULT_LIVER_CANCER_SUBCLASS_G6_UP	1.67	0.041	0.031
CHIANG_LIVER_CANCER_SUBCLASS_POLYSOMY7_UP	1.57	0.048	0.057
BOYAULT_LIVER_CANCER_SUBCLASS_G3_DN	1.87	>0.0001	0.03
BOYAULT_LIVER_CANCER_SUBCLASS_G12_DN	1.68	>0.0001	0.03
YAMASHITA_LIVER_CANCER_STEM_CELL_DN	1.65	0.008	0.032
YAMASHITA_LIVER_CANCER_WITH_EPCAM_DN	1.60	0.01	0.051
CHIANG_LIVER_CANCER_SUBCLASS_POLYSOMY7_UP	1.57	0.048	0.057
CHIANG_LIVER_CANCER_SUBCLASS_PROLIFERATION_UP	-1.69	>0.0001	0.228
VILLANUEVA_LIVER_CANCER_KRT19_UP	-1.72	0.002	0.249

Given the association of NAA40 with the proliferation class of LIHC, we then examined the expression of genes relating to the cell cycle for all liver cancer samples falling within each of the two groups High- and Low-NAA40. Comparison of expression levels for the 125 genes allocated in the KEGG cell cycle category revealed clear differences between the two groups, most prominently the higher expression for a number of cell-cycle genes correlating with the high NAA40 expression group (**Figure 3E**; larger version of the heatmap can be seen in **Figure S4**). A prominent cluster of upregulated genes in High-NAA40 group (marked as group A on heatmap) was identified which included cell division-stimulating genes such as CCNB1 and CCNB2 as well as multiple MCM family genes. In contrast, a cluster of upregulated genes in the Low-NAA40 group (marked as group B) included growth arresting genes such as GADD45A and GADD45B (**Figure 3E**).

Finally, to confirm the validity of our findings we also repeated our analysis in an independent liver cancer patient cohort, GSE112790 (40). Similar to the TCGA cohort, in this second dataset NAA40 was significantly upregulated when comparing normal liver to primary LIHC samples, with the top quartile showing much greater NAA40 expression than the bottom quartile (**Figure S5**). By performing GSEA we again found a number of liver-related genesets to be enriched in both the High and Low NAA40 quartile groups (**Supplementary Tables 3, 4**). Consistent with the above results high NAA40 expressing tumour samples were enriched for gene signatures of more aggressive and proliferative tumour subtypes as well as worse prognosis (**Figures S5A–D** and **Supplementary Tables 3, 4**).

It is interesting to noted that in both the TCGA and the independent LIHC cohort genesets relating to liver differentiation and development were suppressed in the High-NAA40 groups as it would be expected based on the above observations (**Figure 2**). Examples of these genesets include (1) CAIRO_Liver_Development_DN which represents a mouse-derived liver development gene signature, 2) SU_Liver genes identified as having high expression specifically in the liver in a compendium of 91 human and mouse tissues, and 3) HSIAO_liver_specific_genes which are a list of liver selective genes based on a compendium of 19 human samples. Altogether, these findings demonstrate that NAA40 upregulation is prominent in poorly-differentiated aggressive LIHC subtypes.

To summarise, GSEA found an enrichment of NAA40 in genesets associated with the proliferation class of LIHC, while lower expression of the enzyme in iCluster 2 is consistent with its lower levels in the non-proliferating class (**Figures 3A, C, D**).

### High NAA40 Expression in Liver Cancer Correlates With P53-Inactivation

**Figure 4:**
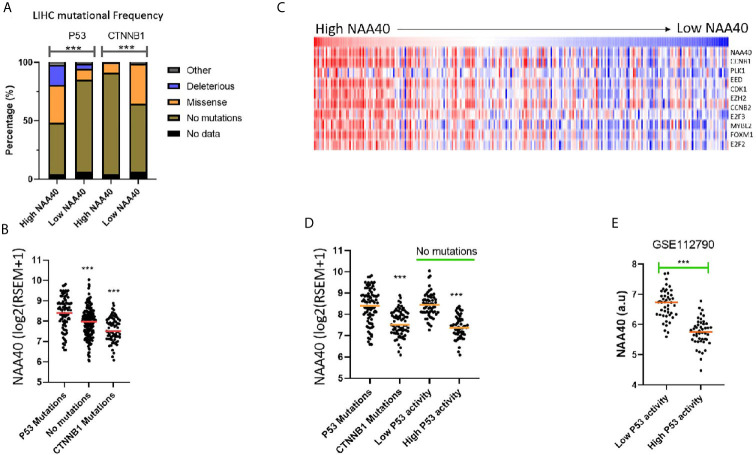
NAA40 upregulation coincides with inactivation of P53 in LIHC **(A)** Stacked bar charts displaying frequencies of TP53 and CTNNB1 mutations in High and Low NAA40 LIHC groups; **(B)** Expression of NAA40 in TCGA LIHC tumours with mutations of TP53, CTNNB1, or neither of the two genes; **(C)** Heatmap comparing expression of NAA40 with ten p53-repressed genes which define a signature indicative of P53 activity; **(D)** Comparison of expression of NAA40 in tumours with P53 mutations, CTNNB1 mutations, and in tumours with no mutations in either of these two genes but divided into top and bottom quartiles of p53 activity according to p53-repressed gene signature; **(E)** Comparison of NAA40 levels in an independent LIHC cohort (GSE112790) demarcated into top and bottom quartiles based on signature of P53 repressed genes; In the scatterplots each circle represents an individual sample and orange line depicts the median value ***p < 0.001 Student’s t-test.

Although the NAA40 gene itself is not a common mutational target in LIHC, it remains possible that its transcriptional deregulation could be associated with genetic alterations of other oncogenic or tumour suppressor genes. In line with this notion, the LIHC proliferation class in which NAA40 is highly upregulated is genetically characterised by *P53* mutations, while the non-proliferation class of LIHC which is not significantly associated with NAA40 expression is enriched with *CTNNB1* mutations [**Figure 3A** and (11)]. To determine potential genomic mutations associated with NAA40 expression in LIHC, we compared the mutational frequencies for High and Low NAA40 groups of LIHC tumours. Interestingly, Fischer’s exact t-test found the mutational frequency of *P53* and *CTNNB1* displaying the most significant difference between the two NAA40 expression groups (p.<0.001). Specifically, for *P53* this comparison found close to 50% deleterious/missense mutations in the High-NAA40 group and only 14% in the Low-NAA40 group. In contrast, for *CTNNB1* the mutational frequencies were 9% in the High group and 34% in the Low group (**Figure 4A**). As expected, NAA40 transcript levels were higher in LIHC samples with *P53* mutations than those with *CTNNB1* by about 1.7-fold on average. LIHC samples that do not have mutations affecting either of those two genes showed intermediate NAA40 expression levels (**Figure 4B**). Consequently, for LIHC a clear inverse pattern of mutation frequency was observed for *P53* and *CTNNB1* in tumours with high and low NAA40 expression.

To investigate further whether the link between p53 activity and NAA40 expression involves direct transcriptional regulation we then examined available transcriptomic studies from studies targeting P53 in liver cells. Activation of P53 with Nutlin treatment in the HepG2 hepatoma cell line caused a decrease in NAA40 levels compared to DMSO treatment (0.7-fold difference between two groups, 2 samples per group), which could be reversed with treatment with shRNA against P53 but not control hairpin (GSE30137, **Figure S6A**). Acute knockout of mouse liver P53 using an adenoviruses system achieving 80% decrease was also associated with a 1.3 fold increase in NAA40 transcript (GSE81226, **Figure S6B**). Finally, comparison of NAA40 levels in non-cancer liver tissue, liver tumour tissue caused by diethylnitrosamine (DEN) treatment and having a wildtype P53, and tumours associated with P53 liver-specific knockout, showed a clear increase in NAA40 levels only in the later model (GSE34760, **Figure S6C**).

It is important to note that in LIHC, as well as other cancers, P53-mediated processes can also be functionally inactivated even when the *P53* gene itself is not mutated through alternative mechanisms including mutation of MDM proteins, dysregulation of microRNAs, as well as action of the hepatitis viruses (41, 42). To investigate NAA40 expression in LIHC samples with alternative P53 inactivation, we utilised a previously defined 10-gene signature of P53 repressed genes whose expression is indicative of P53 activity (30). It was interesting to note that in the TCGA LIHC dataset, NAA40 expression was clearly correlating with the expression of these 10 genes (**Figure 4C**). Even more remarkably, tumours with low P53 activity determined by this 10-gene signature were also associated with high NAA40 expression in the TCGA and an independent LIHC dataset (GSE1127960) (**Figures 4D, E**), analogous to the samples carrying P53 mutations. In conclusion, our analysis indicates a novel connection between P53 deficiency and deregulation of NAA40 in LIHC.

### NAA40 Upregulation in Liver Cancer Correlates With Worse Survival of Patients With P53 Inactivation

**Figure 5:**
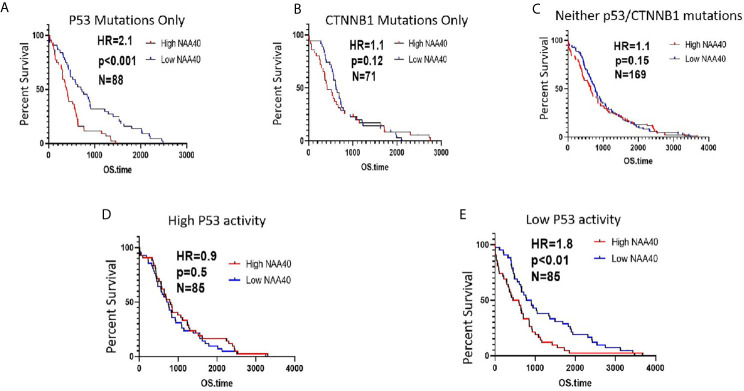
High NAA40 expression predicts worse survival for LIHC patients with inactivated P53 **(A–C)** Kaplan-Meier survival curves for LIHC patients in the TCGA study carrying *P53* mutations, *CTNNB1* mutations or no mutations on either of the two genes divided according to median NAA40 expression; **(D, E)** Kaplan-Meier plots for LIHC samples with no registered P53 mutations that were divided into two groups according to P53 activity using a previously described 10-gene signature. Hazard ratio (HR) and p values were calculated using the log-rank method.

Based on the above findings, we next considered whether the survival of patients carrying *P53* mutations is associated with NAA40 expression. In agreement with the above revealed connection, high NAA40 expression is indicative of worse survival in patients with *P53* mutations, but not in patients carrying *CTNNB1* mutations or lacking mutations in either of the two genes (**Figures 5A–C**). To further support this correlation, we then performed similar analysis in patients with low and high P53 activity as defined using the 10-gene signature of P53 repressed genes (same as **Figure 4C**). Consistently, high NAA40 was significantly associated with worse survival in patients with low but not high P53 activity in the TCGA LIHC cohort (**Figures 5D, E**). Thus, demarcation of LIHC samples according to P53 activity results in similar patterns as in cases with P53 mutations.

To validate the identified associations of NAA40 with survival we additionally examined two independent LIHC cohorts containing transcriptomic and survival data, GSE14520 and GSE 54236. As these two studies did not examine genetic mutations, the functionality of P53 was assessed using the same 10-gene signature of repressed genes as described before. Notably, in both datasets we again found the pattern of significantly higher NAA40 levels in samples characterised as having low P53 activity (**Figure S7A**). For the GSE14520 cohort although not reaching statistical significance, clearly the trend was in the same direction of NAA40 being associated with significance in low P53 activity samples (N=110, HR=1.4 and p=0.1) compared to samples with high P53 activity (N=110, HR=1, p=1.0) (**Figure S7B**). In the smaller GSE54236 cohort NAA40 was significantly associated with survival in the low activity P53 samples (N=39, HR=3.1, p<0.001) but not in the high activity samples (N=39, HR=1.02, p=0.92) (**Figure S7C**).

To summarise, a common trend across three independent LIHC cohorts is that NAA40 expression is associated with worse survival specifically in tumours with low P53 activity.

## Discussion

Our analysis from multiple independent transcriptomic datasets here and previously (27) concur in a consistent trend of NAA40 upregulation in LIHC compared with normal liver and its association with worse survival. These findings are in contrast to Liu et al., who had reported the downregulation of NAA40 in LIHC (43). However, this preliminary analysis of Liu et al, was based on the examination of a small set of nine pairs of tumour and adjacent normal tissues. Therefore, we consider that our analysis of transcriptomic datasets from hundreds of patient samples strongly support the increased levels of NAA40 in LIHC and a potential oncogenic function. In this study, we have also shown for the first time that the NAA40 transcriptional upregulation in LIHC is an early event in LIHC and not dependent on any specific environmental agent (**Figure 1**). Moreover, it was also shown that NAA40 expression is correlated with loss of hepatic differentiation, more proliferative and aggressive tumours, and a worse prognosis (**Figures 2**, **3**). In addition, we found that NAA40 deregulation and association with survival is connected to inactivation of P53 through mutations or other deactivating mechanisms (**Figures 4**, **5** and **S7**).

Although transcript levels of NAA40 have been previously reported to be increased in several cancer types (27, 28, 34), this is the first time that its transcriptional deregulation has been linked to a specific genetic mutation i.e. P53. Given that loss of P53 functionality is a prominent event in the majority of cancer types (44), it is therefore possible that P53-deficiency is involved with the deregulation of NAA40 in other cancer types as well. It is worth noting that we did not find evidence for any putative P53 binding sites using PhysBinder (45) or PROMO (46) software tools in the NAA40 promoter region (2000 base pairs upstream of NAA40) or for direct P53 binding to the promoter of the gene in the ENCODE datasets (47). Mutations of P53 can affect gene expression either due to loss-of-function of the tumour suppressor or by gain-of-function of the mutant protein. In relation to its connection with NAA40 the former possibility appears as the most likely outcome, since this NAT displayed increased expression even when P53 had reduced activity in samples with no accompanying P53 mutations. Hence, it is currently unclear whether loss of P53 influences NAA40 levels through direct or indirect mechanisms.

A limitation of this study is that we did not examine the effects of manipulating NAA40 directly in suitable *in vitro* or *in vivo* liver cell models. Consequently, it is possible that the observed upregulation of NAA40 is a passive consequence of liver aggressiveness and loss of hepatic differentiation rather than having a causal role in hepatocarcinogenesis. An important future task will be to perform such experiments in order to clarify the biological consequences of altered NAA40 levels in LIHC. Of particular interest will be to identify the potential genes and pathways through which NAA40 can potentially affect carcinogenesis and survival in LIHC patients possessing inactivated P53. Unfortunately LIHC with alterations of P53 have a particularly dismal disease progression and prognosis (48), rendering research into factors that can modulate this pathway of particular relevance. Finally, beyond the unassailable role of P53 in carcinogenesis, it has also been appreciated that this protein is involved in important aspects of normal liver biology such as regeneration after injury (49) and metabolic response to starvation (50). Therefore, the P53-NAA40 link could be of biological significance not only in tumour but also in normal liver.

Molecular signatures in cancer have multiple potential uses including improved diagnosis, prognosis, and patient stratification for treatment. A number of studies in recent years have aimed toward identifying molecular signatures for LIHC, for examples see (51–54). In general, such studies improve their potency through the inclusion of multiple variates generated by one or more -omic technological platforms. Using NAA40 expression as a univariate prognostic survival marker for the TCGA LIHC cohort revealed prognostic ability (HR=1.67; p=0.003) that is similar or better than other candidate genes when examined by themselves [e.g. FCN3, CLEC1B, and PRC1 in (54)] although not as good as multi-variant classifiers [e.g., (51, 53)]. Further work is needed to validate the prognostic ability of NAA40 in multiple independent LIHC and to examine whether it can be integrated with other multi-variate predictive and prognostic signatures. An interesting possibility is that the prognostic ability of NAA40 could potentially prove to be of particular relevance in the LIHC subset of patients with P53 mutations.

In conclusion, previously published work has suggested oncogenic functions for NAT family members such as NAA10 (55) and NAA20 (23, 24, 56) in LIHC. Indeed, these two NATs were the second and third most upregulated in the comparison of LIHC and adjacent normal tissue in the TCGA (**Table 2**). However, in our analysis NAA40 emerges as potentially the NAT with the most important role in LIHC based on its higher upregulation and association with worse disease prognosis. Further investigations into the genes and pathways regulated by NAA40 in LIHC and the potential consequences of targeting this enzyme for tumour growth and survival are therefore warranted.

## Data Availability Statement

Publicly available datasets were analysed in this study. This data can be found here: TCGA (https://www.cancer.gov/about-nci/organization/ccg/research/structural-genomics/tcga) GEO omnibus https://www.ncbi.nlm.nih.gov/geo/ (GSE112790, GSE89632, GSE49541, GSE84044).

## Author Contributions

Conceptualization—CK and AK. Methodology, CK. Formal Analysis, CK. Investigation, CK. Data Curation, CK. Writing—Original Draft Preparation, CK. Writing—Review and Editing, CK and AK. Supervision, AK. Project Administration, CK and AK. Funding Acquisition, CK and AK. All authors contributed to the article and approved the submitted version.

## Funding

This work was co-funded by the European Regional Development Fund and the Republic of Cyprus through the Research and Innovation Foundation (project: EXCELLENCE/0918/0081). Work in the AK lab is also supported by funding from the Cyprus Cancer Research Institute’s (C.C.R.I.) Bridges in research excellence (CCRI_2020_FUN_001) under Funding agreement No CCRI_2021_FA_LE_106.

## Conflict of Interest

The authors declare that the research was conducted in the absence of any commercial or financial relationships that could be construed as a potential conflict of interest.
