# Supplementary material for: Identification of NAA40 as a Potential Prognostic Marker for Aggressive Liver Cancer Subtypes
Source: Front Oncol. 2021 Jun 2;11:691950. doi: 10.3389/fonc.2021.691950 (PMC8208081; doi:10.3389/fonc.2021.691950)
Supplement: Supplementary file 1 [file DataSheet_1.docx]

**Supplementary Tables**

**Suppl. Table 1**: Genesets significantly enriched in High NAA40 quartile of TCGA LIHC

| **GS DETAILS** | **GS SIZE** | **ES** | **NES** | **NOM p-val** | **FDR q-val** |
| --- | --- | --- | --- | --- | --- |
| CHIANG_LIVER_CANCER_SUBCLASS_PROLIFERATION_UP | 176 | -0.8 | -1.7 | 0 | 0.228 |
| GSE25085_FETAL_LIVER_VS_ADULT_BM_SP4_THYMIC_IMPLANT_UP | 191 | -0.4 | -1.8 | 0.016 | 0.247 |
| VILLANUEVA_LIVER_CANCER_KRT19_UP | 176 | -0.7 | -1.7 | 0.002 | 0.249 |

Significance threshold set as Nominal p.val<0.05 and FDR q-value <0.25

**Suppl. Table 2**: Genesets significantly enriched in Low NAA40 quartile of TCGA LIHC

| **GS DETAILS** | **GS SIZE** | **ES** | **NES** | **NOM p-val** | **FDR q-val** |
| --- | --- | --- | --- | --- | --- |
| ACEVEDO_NORMAL_TISSUE_ADJACENT_TO_LIVER_TUMOR_DN | 345 | 0.6 | 2.09 | 0.002 | 0.005 |
| HOSHIDA_LIVER_CANCER_SUBCLASS_S3 | 266 | 0.82 | 1.98 | 0 | 0.013 |
| IIZUKA_LIVER_CANCER_PROGRESSION_G2_G3_UP | 27 | 0.74 | 1.79 | 0.002 | 0.016 |
| BOYAULT_LIVER_CANCER_SUBCLASS_G1_DN | 40 | 0.73 | 1.79 | 0.008 | 0.017 |
| WOO_LIVER_CANCER_RECURRENCE_DN | 78 | 0.89 | 1.8 | 0 | 0.018 |
| LEE_LIVER_CANCER_CIPROFIBRATE_DN | 66 | 0.69 | 1.78 | 0.002 | 0.018 |
| ANDERSEN_LIVER_CANCER_KRT19_DN | 74 | 0.88 | 1.83 | 0 | 0.019 |
| LEE_LIVER_CANCER_DENA_DN | 74 | 0.73 | 1.81 | 0 | 0.019 |
| LEE_LIVER_CANCER_ACOX1_DN | 65 | 0.73 | 1.81 | 0 | 0.019 |
| LEE_LIVER_CANCER_MYC_E2F1_DN | 63 | 0.73 | 1.8 | 0 | 0.019 |
| LEE_LIVER_CANCER_MYC_TGFA_DN | 64 | 0.72 | 1.76 | 0 | 0.019 |
| LEE_LIVER_CANCER_MYC_DN | 62 | 0.63 | 1.76 | 0.004 | 0.02 |
| BOYAULT_LIVER_CANCER_SUBCLASS_G123_DN | 51 | 0.89 | 1.75 | 0 | 0.02 |
| WENG_POR_TARGETS_LIVER_UP | 37 | 0.64 | 1.75 | 0.013 | 0.021 |
| HOSHIDA_LIVER_CANCER_SURVIVAL_DN | 111 | 0.57 | 1.74 | 0.016 | 0.021 |
| LEE_LIVER_CANCER_E2F1_DN | 63 | 0.71 | 1.84 | 0 | 0.022 |
| HSIAO_LIVER_SPECIFIC_GENES | 248 | 0.86 | 1.83 | 0 | 0.022 |
| CAIRO_LIVER_DEVELOPMENT_DN | 215 | 0.62 | 1.73 | 0.008 | 0.022 |
| CHIANG_LIVER_CANCER_SUBCLASS_PROLIFERATION_DN | 177 | 0.91 | 1.72 | 0 | 0.022 |
| CHIANG_LIVER_CANCER_SUBCLASS_CTNNB1_UP | 172 | 0.79 | 1.72 | 0.02 | 0.023 |
| ACEVEDO_LIVER_TUMOR_VS_NORMAL_ADJACENT_TISSUE_DN | 271 | 0.56 | 1.71 | 0.012 | 0.024 |

Significance threshold set as Nominal p.val<0.05 and FDR q-value <0.25

**Suppl. Table 3**: Genesets significantly enriched in High NAA40 quartile of GSE112790 cohort

| **GS DETAILS** | **GS size** | **ES** | **NES** | **NOM p-val** | **FDR q-val** |
| --- | --- | --- | --- | --- | --- |
| BOYAULT_LIVER_CANCER_SUBCLASS_G3_UP | 187 | 0.72 | 1.66 | 0.002 | 0.067 |
| VILLANUEVA_LIVER_CANCER_KRT19_UP | 177 | 0.8 | 1.69 | 0 | 0.07 |
| LEE_LIVER_CANCER_SURVIVAL_DN | 176 | 0.71 | 1.71 | 0 | 0.078 |
| ANDERSEN_LIVER_CANCER_KRT19_UP | 34 | 0.63 | 1.66 | 0.014 | 0.082 |
| BOYAULT_LIVER_CANCER_SUBCLASS_G1_UP | 114 | 0.64 | 1.56 | 0.037 | 0.106 |
| BOYAULT_LIVER_CANCER_SUBCLASS_G23_UP | 51 | 0.84 | 1.61 | 0 | 0.107 |
| KAPOSI_LIVER_CANCER_MET_UP | 18 | 0.73 | 1.57 | 0.015 | 0.118 |
| CHIANG_LIVER_CANCER_SUBCLASS_PROLIFERATION_UP | 176 | 0.91 | 1.58 | 0 | 0.124 |
| BOYAULT_LIVER_CANCER_SUBCLASS_G123_UP | 47 | 0.81 | 1.59 | 0.002 | 0.127 |
| GSE25085_FETAL_LIVER_VS_ADULT_BM_SP4_THYMIC_IMPLANT_DN | 193 | 0.66 | 1.71 | 0.006 | 0.15 |
| SHETH_LIVER_CANCER_VS_TXNIP_LOSS_PAM1 | 248 | 0.44 | 1.49 | 0.021 | 0.15 |
| GSE5503_LIVER_DC_VS_MLN_DC_ACTIVATED_ALLOGENIC_TCELL_UP | 194 | 0.4 | 1.49 | 0.006 | 0.156 |
| GSE369_SOCS3_KO_VS_IFNG_KO_LIVER_DN | 194 | 0.48 | 1.49 | 0.015 | 0.163 |
| HOSHIDA_LIVER_CANCER_SUBCLASS_S2 | 114 | 0.53 | 1.5 | 0.128 | 0.173 |
| CHIANG_LIVER_CANCER_SUBCLASS_POLYSOMY7_DN | 25 | 0.76 | 1.47 | 0.041 | 0.176 |
| YAMASHITA_LIVER_CANCER_WITH_EPCAM_UP | 51 | 0.59 | 1.5 | 0.031 | 0.182 |
| KIM_LIVER_CANCER_POOR_SURVIVAL_UP | 22 | 0.8 | 1.46 | 0.047 | 0.183 |
| ACEVEDO_LIVER_CANCER_WITH_H3K9ME3_DN | 89 | 0.46 | 1.42 | 0.036 | 0.194 |
| GSE5503_LIVER_DC_VS_SPLEEN_DC_ACTIVATED_ALLOGENIC_TCELL_UP | 193 | 0.37 | 1.42 | 0.032 | 0.195 |
| SERVITJA_LIVER_HNF1A_TARGETS_UP | 129 | 0.54 | 1.44 | 0.027 | 0.209 |

Significance threshold set as Nominal p.val<0.05 and FDR q-value <0.25

**Suppl. Table 4**: Genesets significantly enriched in Low NAA40 quartile of GSE112790 cohort

| **GS DETAILS** | **GS size** | **ES** | **NES** | **NOM p-val** | **FDR q-val** |
| --- | --- | --- | --- | --- | --- |
| HOSHIDA_LIVER_CANCER_SUBCLASS_S3 | 263 | -0.8 | -1.83 | 0 | 0.005 |
| IIZUKA_LIVER_CANCER_PROGRESSION_G2_G3_UP | 27 | -0.78 | -1.84 | 0 | 0.009 |
| BOYAULT_LIVER_CANCER_SUBCLASS_G3_DN | 52 | -0.84 | -1.79 | 0 | 0.009 |
| VILLANUEVA_LIVER_CANCER_KRT19_DN | 68 | -0.83 | -1.77 | 0 | 0.011 |
| LEE_LIVER_CANCER_MYC_DN | 61 | -0.71 | -1.76 | 0.004 | 0.011 |
| BOYAULT_LIVER_CANCER_SUBCLASS_G123_DN | 51 | -0.92 | -1.7 | 0 | 0.014 |
| CHIANG_LIVER_CANCER_SUBCLASS_PROLIFERATION_DN | 177 | -0.93 | -1.68 | 0 | 0.014 |
| ANDERSEN_LIVER_CANCER_KRT19_DN | 74 | -0.82 | -1.68 | 0.002 | 0.014 |
| LEE_LIVER_CANCER_CIPROFIBRATE_DN | 66 | -0.72 | -1.66 | 0.002 | 0.014 |
| LEE_LIVER_CANCER_MYC_E2F1_DN | 62 | -0.75 | -1.67 | 0.004 | 0.014 |
| CAIRO_LIVER_DEVELOPMENT_DN | 215 | -0.64 | -1.67 | 0.024 | 0.014 |
| HOSHIDA_LIVER_CANCER_SURVIVAL_DN | 111 | -0.63 | -1.69 | 0.026 | 0.014 |
| CHIANG_LIVER_CANCER_SUBCLASS_UNANNOTATED_UP | 84 | -0.77 | -1.71 | 0.002 | 0.015 |
| WOO_LIVER_CANCER_RECURRENCE_DN | 78 | -0.85 | -1.69 | 0.002 | 0.015 |
| LEE_LIVER_CANCER_SURVIVAL_UP | 179 | -0.81 | -1.72 | 0.004 | 0.015 |
| LEE_LIVER_CANCER_ACOX1_DN | 64 | -0.74 | -1.7 | 0.006 | 0.015 |
| OHGUCHI_LIVER_HNF4A_TARGETS_DN | 156 | -0.76 | -1.68 | 0.006 | 0.015 |
| WENG_POR_TARGETS_LIVER_DN | 22 | -0.78 | -1.67 | 0.006 | 0.015 |
| SHETH_LIVER_CANCER_VS_TXNIP_LOSS_PAM4 | 272 | -0.62 | -1.72 | 0.008 | 0.015 |
| KIM_LIVER_CANCER_POOR_SURVIVAL_DN | 43 | -0.89 | -1.65 | 0 | 0.016 |
| HSIAO_LIVER_SPECIFIC_GENES | 246 | -0.83 | -1.69 | 0.002 | 0.016 |
| LEE_LIVER_CANCER_E2F1_DN | 62 | -0.69 | -1.63 | 0.006 | 0.019 |
| LEE_LIVER_CANCER_DENA_DN | 74 | -0.74 | -1.63 | 0.01 | 0.019 |
| BOYAULT_LIVER_CANCER_SUBCLASS_G1_DN | 40 | -0.72 | -1.62 | 0.04 | 0.02 |
| YAMASHITA_LIVER_CANCER_STEM_CELL_DN | 75 | -0.8 | -1.61 | 0.008 | 0.023 |
| CHIANG_LIVER_CANCER_SUBCLASS_CTNNB1_UP | 172 | -0.83 | -1.61 | 0.012 | 0.023 |
| LEE_LIVER_CANCER_MYC_TGFA_DN | 64 | -0.71 | -1.6 | 0.018 | 0.025 |
| SERVITJA_LIVER_HNF1A_TARGETS_DN | 157 | -0.62 | -1.59 | 0.014 | 0.028 |
| WANG_RECURRENT_LIVER_CANCER_DN | 16 | -0.77 | -1.58 | 0.039 | 0.031 |
| SU_LIVER | 57 | -0.86 | -1.55 | 0.01 | 0.038 |
| CHIANG_LIVER_CANCER_SUBCLASS_POLYSOMY7_UP | 75 | -0.8 | -1.54 | 0.011 | 0.042 |
| BOYAULT_LIVER_CANCER_SUBCLASS_G12_DN | 15 | -0.89 | -1.53 | 0.008 | 0.045 |
| MINGUEZ_LIVER_CANCER_VASCULAR_INVASION_DN | 21 | -0.89 | -1.52 | 0.01 | 0.048 |
| ACEVEDO_LIVER_TUMOR_VS_NORMAL_ADJACENT_TISSUE_DN | 268 | -0.6 | -1.51 | 0.044 | 0.054 |
| HOSHIDA_LIVER_CANCER_LATE_RECURRENCE_DN | 69 | -0.51 | -1.48 | 0.033 | 0.072 |

Significance threshold set as Nominal p.val<0.05 and FDR q-value <0.25

**Supplementary Figures**

**Fig.S1** Frequency of mutations (copy number variants or exonic mutations) for NAT genes in the TCGA pan-cancer LIHC project detected according to cBiolportal tool (https://www.cbioportal.org/).

**
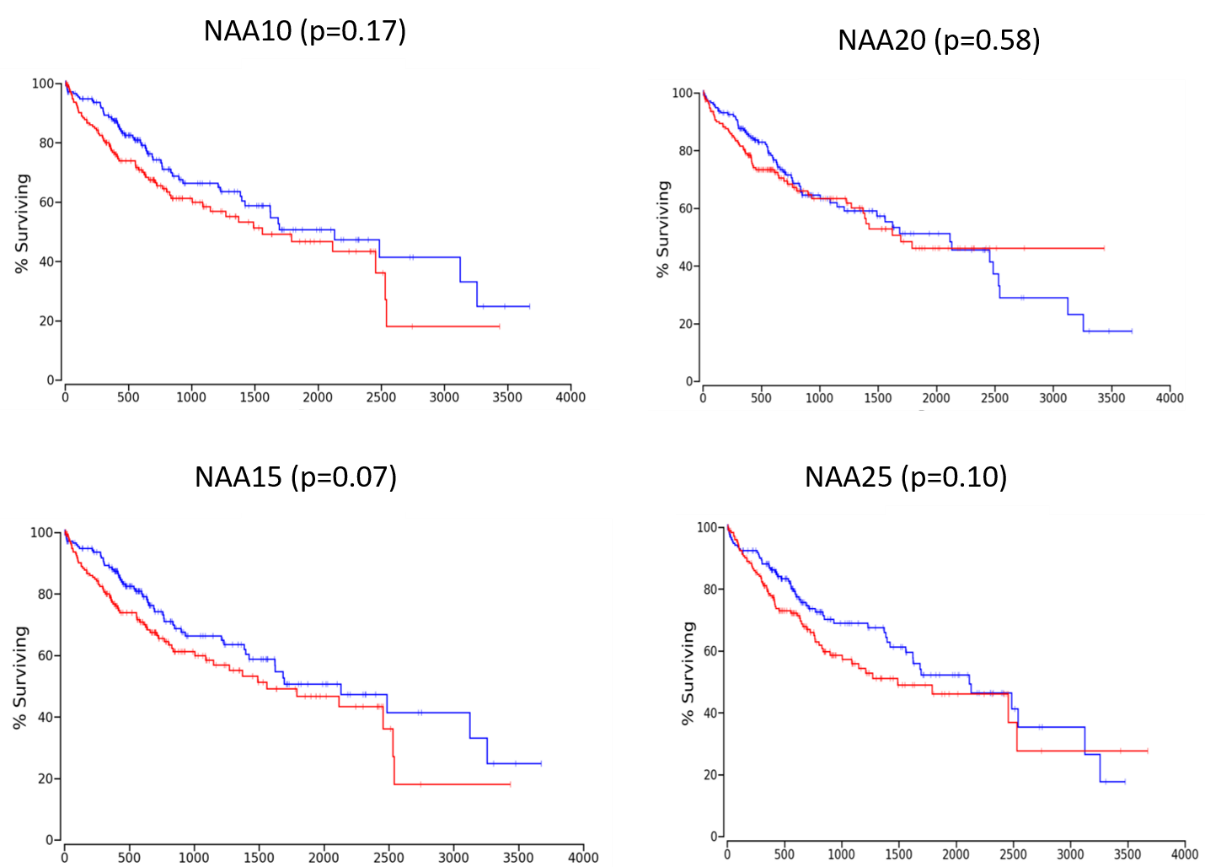
**

**
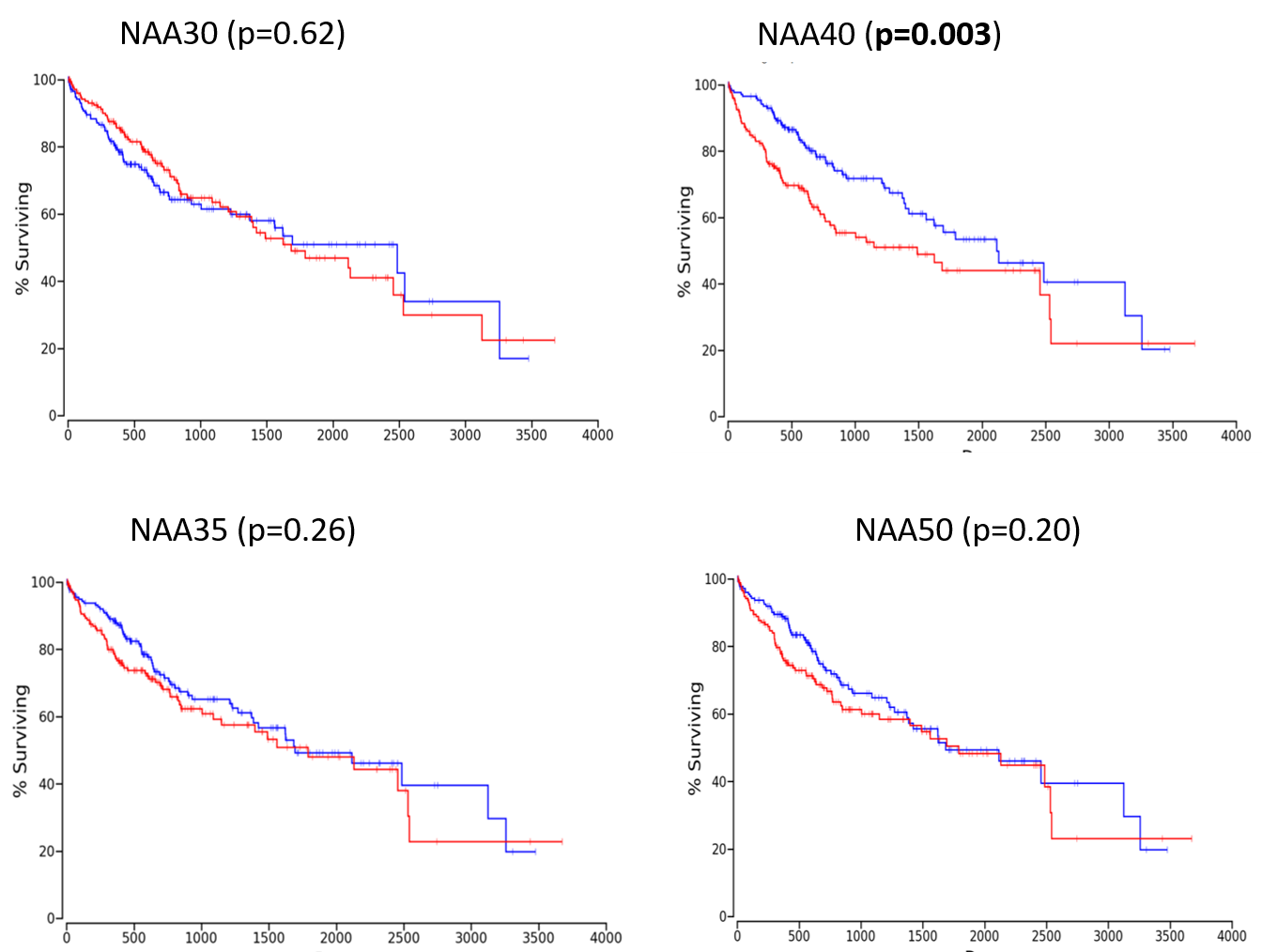
**

**
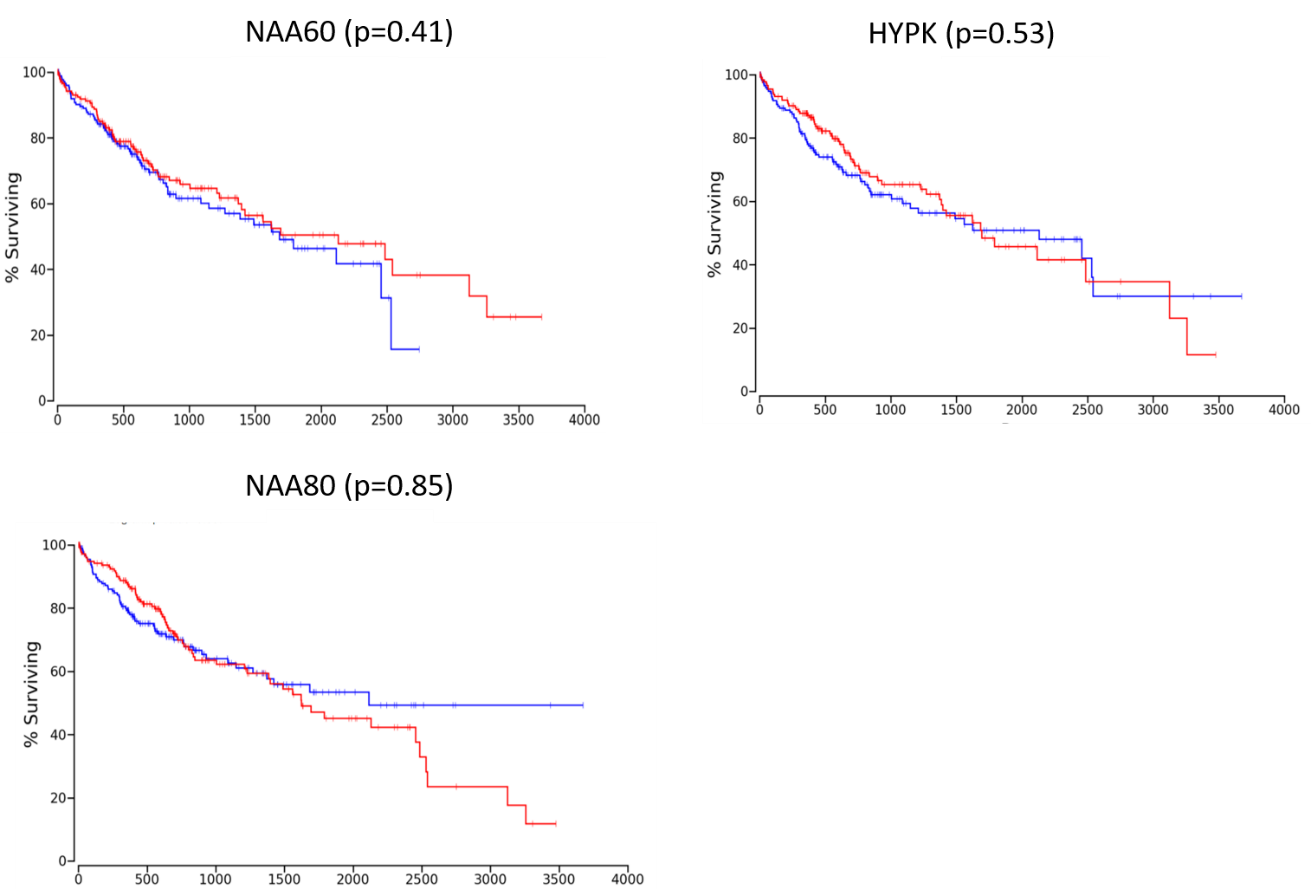
**

**Fig.S2** Kaplan Meier curves showing association of 11 NAT family members with survival of LIHC patients, derived using the Oncolnc tool (<http://www.oncolnc.org/>). Y-axis displays % of surviving patients and the x-axis in the number of days. Samples were divided according to median NAT gene expression, with the blue line indicating samples with low NAT gene expression and the red line indicating high NAT gene expression (N=180 samples each group). The p.values were estimated using the logrank method. Only NAA40 was significantly associated with survival of LIHC patients (p<0.05).

**Fig.S3** Comparison of NAA40 mRNA and protein levels across 14 liver cell lines with available paired data from DepMap project (<https://depmap.org/portal/>). Each circle represents an individual cell line, the line shown indicates linear regression, and Pearson’s correlation was calculated.


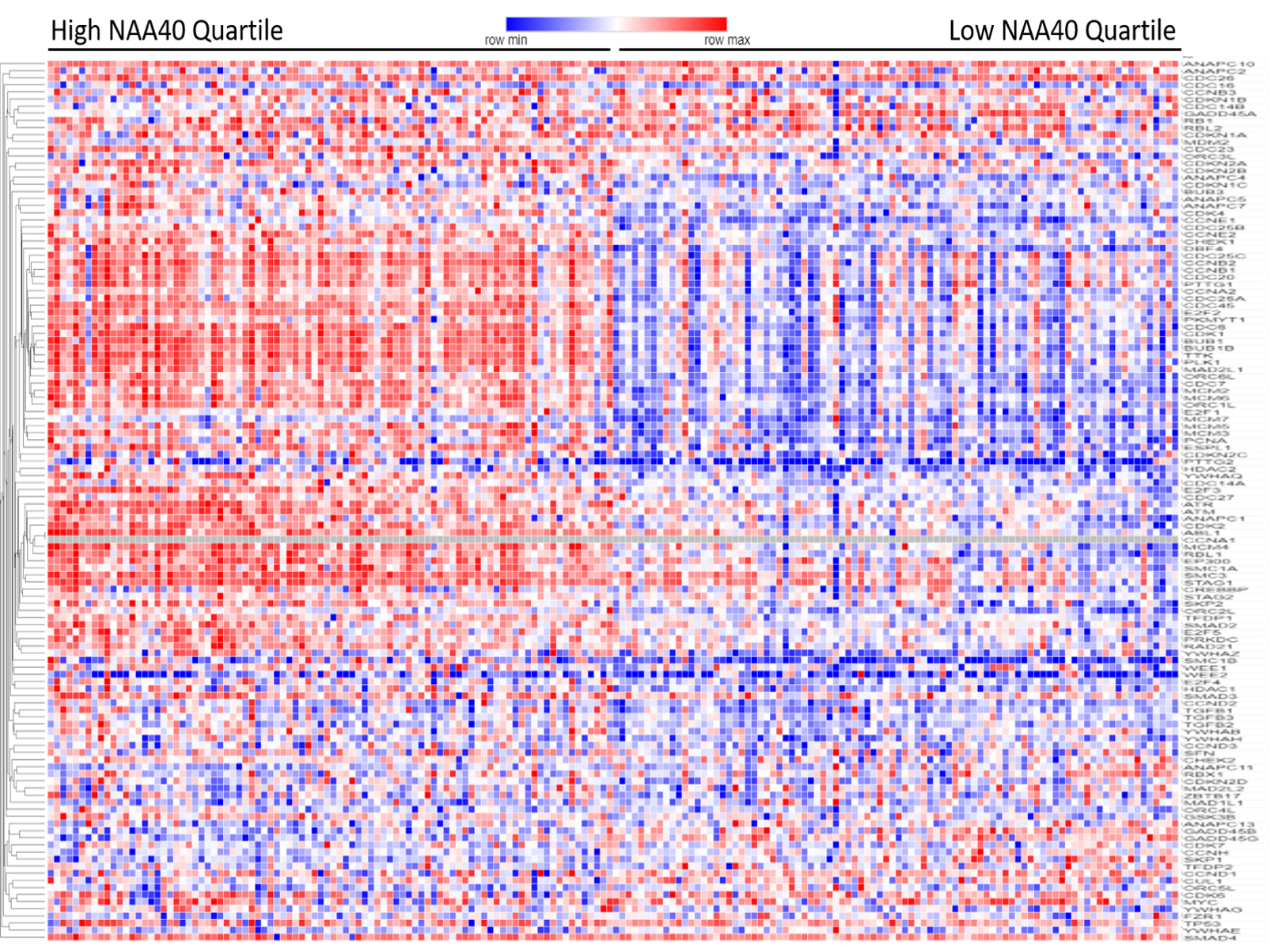


**Fig.S4** Heatmap of cell cycle gene expression in High and Low NAA40 quartiles in TCGA LIHC. Each row represents a cell cycle gene and each column an LIHC TCGA sample.


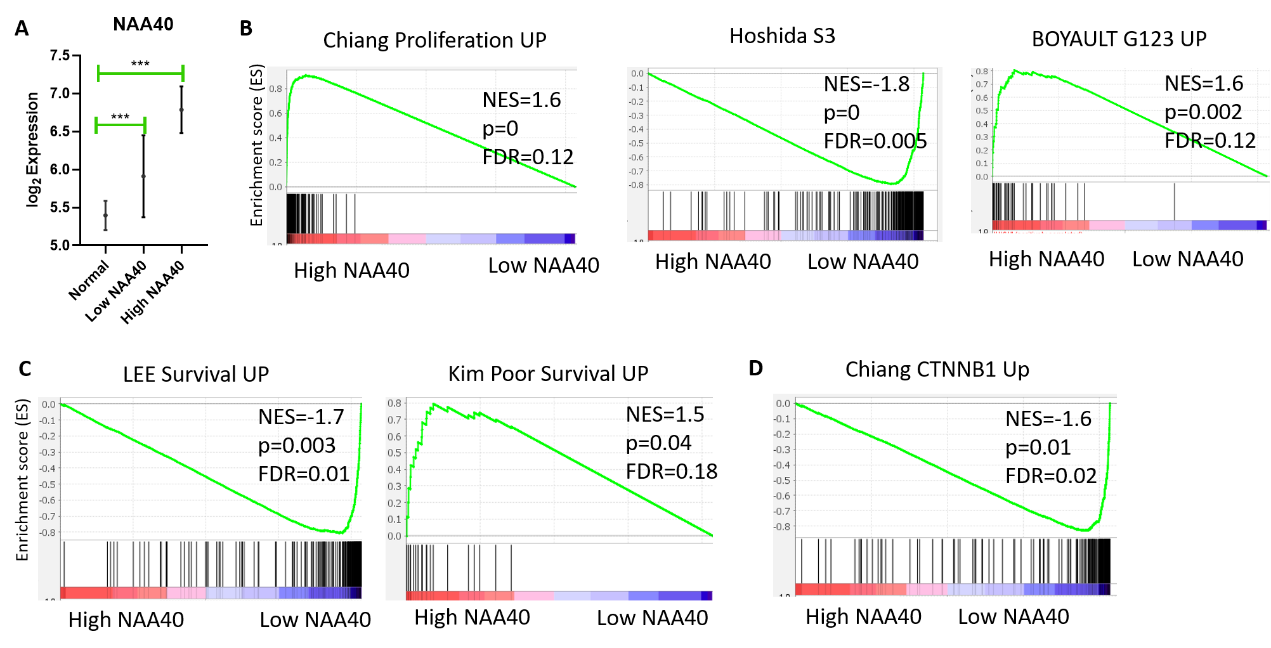


**Fig.S5** Association of NAA40 with more aggressive, proliferating liver genesets in the independent liver cancer cohort GSE112790. (**A**) NAA40 expression in normal liver and in low and high NAA40 quartiles; (**B**) Selected GSEA plots associated with LIHC subtypes; (**C**) selected GSEA genesets associated with LIHC survival; (**D**) Enriched Chiang’s CTNNB1 geneset.


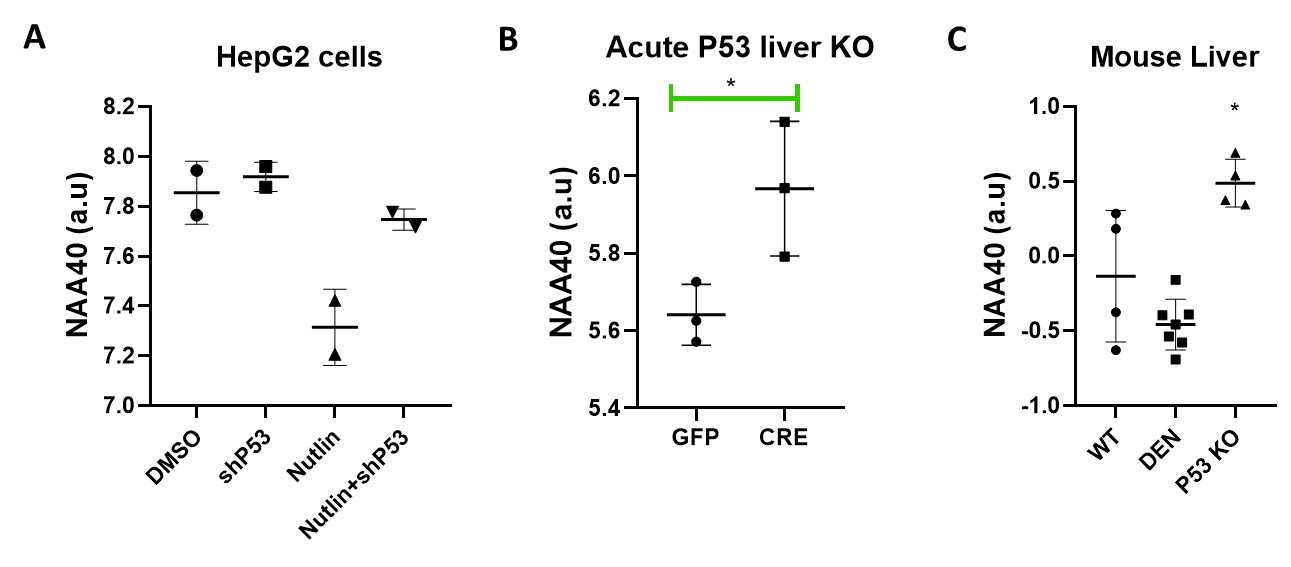


**Fig.S6** Examination of effect of P53 manipulation on NAA40 expression in (**A**) HepG2 cells treated with DMSO or P53 activator Nutlin and shRNA against P53; (**B**) Adenoviral transfection of mouse liver with GFP control or CRE which causes acute P53 knockout; (**C**) Levels of NAA40 in wildtype (WT) non cancer liver tissue, liver cancers generated in P53 KO mouse modes or in P53 wild-type tumours induced by treatment with the DEN carcinogen. Each symbol in graphs represents one sample, * p<0.05 by Student’s t-test or ANOVA.


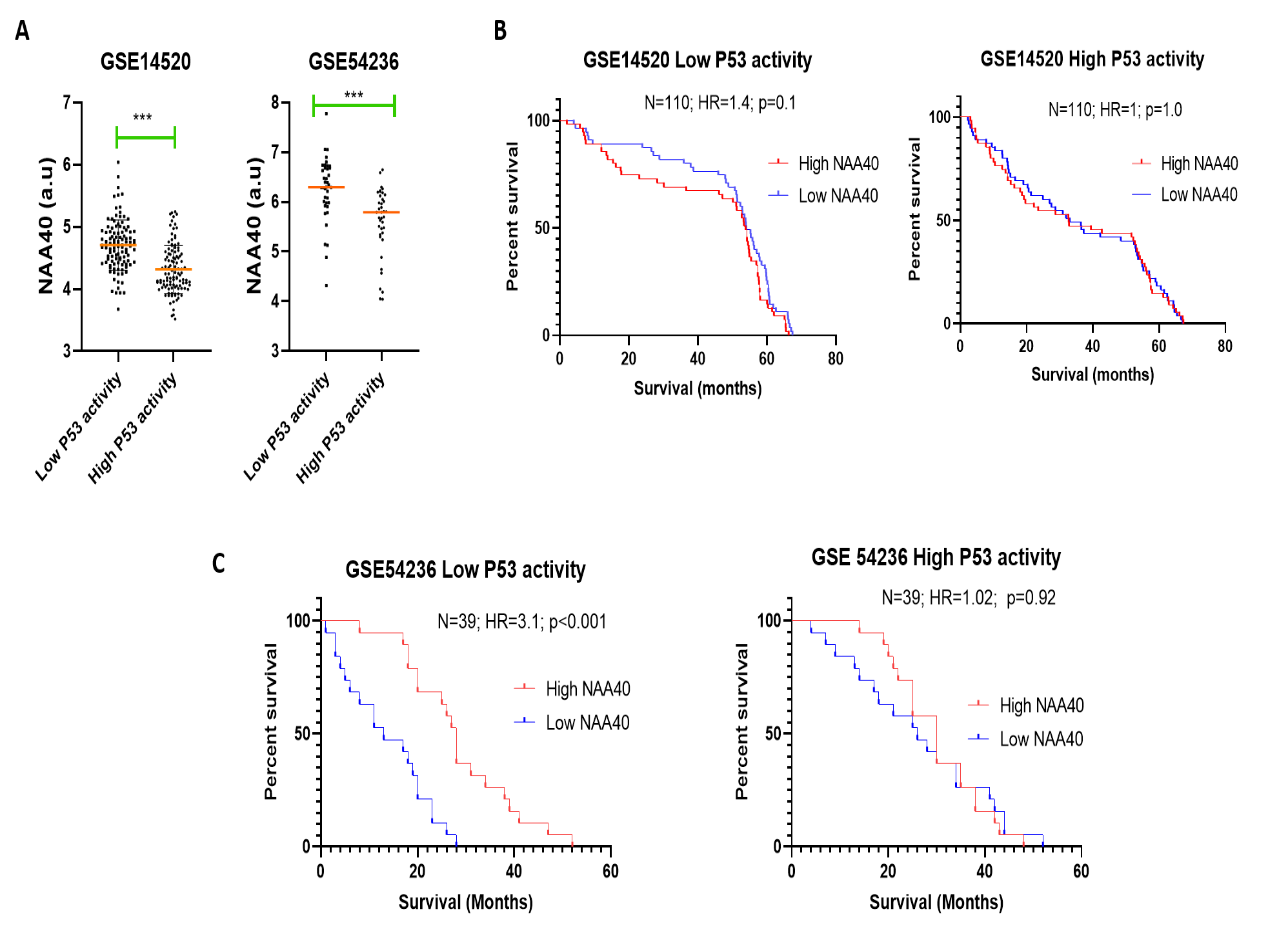


**Fig.S7** Examination of the association between NAA40 expression and survival in LIHC patients with low or high P53 activity (**A**) For two LIHC cohorts P53 activity was evaluated using a 10-gene signature of P53 target genes. For each study then patients where divided into two groups according to this gene signature and NAA40 values were then plotted. Each symbol represents a patient sample, the orange line depicts median expression, statistical significance was tested by Student’s T-test; Kaplan–Meier graphs displaying survival of patients with high or low NAA40 expression in samples with low or high P53 activity in (**B**) GSE14520 cohort and (**C**) GSE54236 cohort. Hazard rations (HR) and statistical significance were calculated using the log-rank method.
